# A new modified Gant-Miwa-Thiersch combined with submucosal and perirectal sclerosant injection procedure for full-thickness rectal prolapse in elderly women: clinical analysis of 34 cases

**DOI:** 10.1186/s12893-021-01284-2

**Published:** 2021-06-05

**Authors:** Jinxi Wang, Huiyu Li, Xiaoming Ma, Gang Du, Jun Ma, Xiaojing Ren, Fang Zhang, Xiushan Dong, Haoliang Zhao, Chongren Ren

**Affiliations:** 1 Shanxi Bethune Hospital, Taiyuan, 030012 People’s Republic of China; 2 Burn Treatment Center of Shanxi Province, Tisco General Hospital, Taiyuan, 030000 People’s Republic of China; 3 Department of Colorectal Surgery, Shanxi Bethune Hospital, Longcheng Street 99, Taiyuan, 030012 People’s Republic of China

**Keywords:** Rectal prolapse, Full-thickness rectal prolapse, Gant-Miwa-Thiersch procedure, nmGMTSI, Recurrence

## Abstract

**Background:**

Full-thickness rectal prolapse (FTRP) frequently occurs in elderly women, and more than 100 surgical procedures have been proposed to restore FTRP. The Gant-Miwa-Thiersch (GMT) procedure is the most used treatment in China. However, the recurrence rate of FTRP post-GMT, which is as high as 23.8%, is concerning. We described a new modified GMT combined with internal and external rectal sclerosant injection (nmGMTSI) procedure to address this problem.

**Methods:**

The nmGMTSI was performed under spinal anesthesia in 34 frail, elderly female patients with FTRP. The surgical results of FTRP were assessed. Fecal incontinence and constipation were evaluated using the Wexner score, and anal canal rest pressure (ACRP), maximum anal systolic pressure (MASP), anorectal sensation thresholds (AST), and maximum rectal tolerance (MRT) using anorectal manometry preoperatively and postoperatively. The causes of recurrence and complications were analyzed.

**Results:**

All patients were cured according to the clinical cure standard. The perioperative Wexner fecal incontinence score (WFIS) was 10.3 ± 3.31, which became 3.7 ± 2.43 (P < 0.0001) postoperatively. The perioperative ACRP was 2.0 ± 0.56 kPa, which became 8.5 ± 2.25 kPa (P < 0.0001) postoperatively. The perioperative MASP was 4.5 ± 1.16 kPa, which became 18.6 ± 2.50 kPa (P < 0.0001) postoperatively. However, no significant difference was observed between the preoperative and postoperative Wexner constipation scores (WCS) (17.3 ± 2.25 vs. 15.4 ± 2.89, P = 0.1047). The perioperative and postoperative AST were 38.1 ± 5.34 mL and 23.5 ± 3.61 mL, respectively (P = 0.0002). The maximum rectal tolerance (MRT) was 157.1 ± 16.73 mL, which became 121.2 ± 12.45 mL postoperatively (P = 0.0009). The patients developed no serious postoperative complications. The total relapse rate after nmGMTSI was 2.9% in the median two years follow-up period. The most common cause of relapse after nmGMTSI was the removal of infected threads used in the Thiersch procedure.

**Conclusion:**

The benefits of nmGMTSI include low rates of recurrence, complications, and mortality, cost-effectiveness, wide adaptation, minimal invasiveness, and technical simplicity. Hence, it should be considered the first option for the treatment of FTRP in frail elderly women.

## Background

Full-thickness rectal prolapse (FTRP) frequently occurs in older women [1]. Its chief clinical feature is a mass protruding from the anus following defecation. Occasionally, the symptoms of FTRP may occur spontaneously while coughing or weight-bearing. FTRP is a debilitating condition with a complex etiology, with obstetric trauma as the most common, iatrogenic sphincter injury during hemorrhoidectomy and fistula surgery, and external injury to the perineum [2].

More than 100 surgical procedures have been proposed to restore FTRP. The operative procedures for rectal prolapse can be broadly categorized as either abdominal or perineal approaches [3]. Traditionally, the perineal approach has been chosen for older, high-risk patients because of fewer surgical complications, shorter surgery time, and simpler anesthesia. Perineal procedures can be classified into the two following categories: procedures to initiate fibrosis, such as submucosal sclerosant injection, and procedures to shorten the prolapsed rectum, such as Delorme’s operation, Altemeier’s operation, stapled transanal rectal resection (STARR), and Gant-Miwa-Thiersch (GMT). The GMT procedure is the most common treatment in China and Japan [4, 5]. However, the recurrence rate of FTRP after GMT is as high as 23.8% [4].

We described a new modified GMT combined with internal and external rectal sclerosant injection (nmGMTSI) procedure to address this problem. To the best of our knowledge, this is the first reported case of such an approach to treat FTRP, with significant results in frail elderly women.

## Methods

### Patient selection

From January 2016 to December 2018, female patients diagnosed with FTRP based on clinical examination [6] who underwent nmGMTSI at the Colorectal Surgery Unit of Shanxi Bethune Hospital were recruited. Patients were recruited based on the following criteria: age > 65 years; length of rectal prolapse > 4 cm; unfit for general anesthesia with chronic heart failure, chronic obstructive pulmonary disease, chronic renal failure requiring hemodialysis, liver cirrhosis, and other conditions. In contrast, patients with FTRP were excluded based on the following criteria: enterocele or cystocele by bimanual examination; malignant colorectal tumors; life expectancy < 2 years; severe colonic transit dysfunction by colon transfer test; recurrent rectal prolapse; absolute contraindication of surgery by whole blood cell analysis, blood clotting index, hepatic and renal function, and routine fecal tests.

All operations were performed by the same surgical team members of the unit. To guarantee the quality of surgery, the main surgeon of the study had 10 years of working experience at the Colorectal Surgery Unit of Shanxi Bethune Hospital. The surgical results of FTRP were assessed by clinical symptoms and bowel function. The bowel function of all patients in the study was measured for fecal incontinence and constipation using the Wexner score, anal canal rest pressure (ACRP), maximum anal systolic pressure (MASP), anorectal sensation thresholds (AST), and maximum rectal tolerance (MRT) by anorectal manometry before and 24 months after surgery. Patients were followed up every 3, 6, 12, and 24 months in the outpatient clinic.

### Operative procedures

**Fig. 1:**
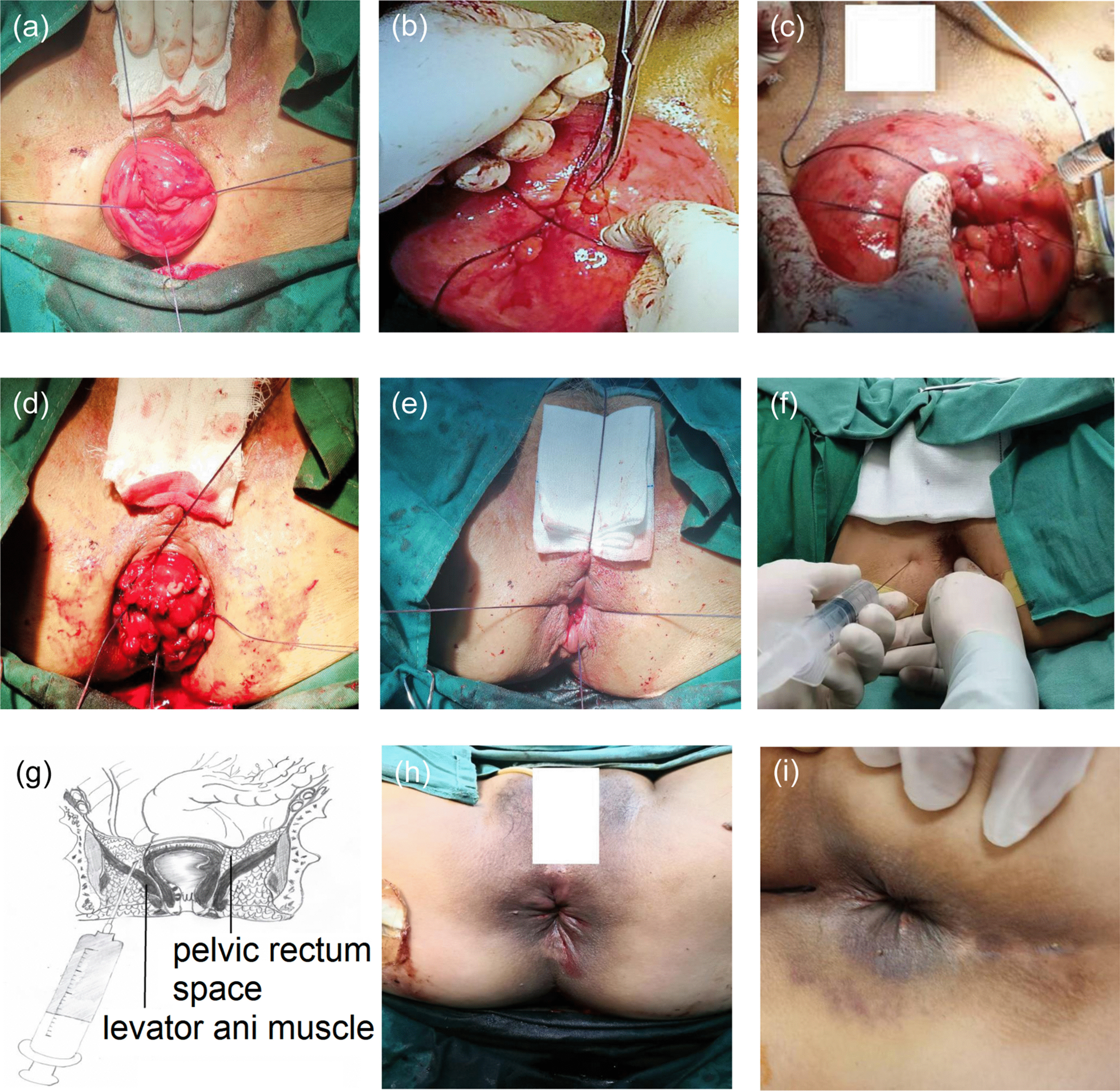
Operative procedure for a new modified Gant-Miwa-Thiersch (GMT) combined with internal and external rectal sclerosant injection (nmGMTSI). **a** The prolapsed rectum was pulled out of the anus completely. Grips of the rectal mucosa were transfixed by a 2/0 Vicryl thread at the 3, 6, 9, and 12 o’clock positions, 5 cm above the surface of the prolapsed mucosa. **b** Grips of rectal prolapsed mucosa were transfixed by silk thread. **c** Sclerosant was injected into the rectal submucosa proximal to mucosal plication. **d** The prolapsed rectal mucosa 1 cm above the dentate line was sutured with the previous uncut Vicryl thread 2/0. **e** The prolapsed rectum was restored completely by tightening the Vicryl thread. **f** Sclerosant was injected into the pelvic rectum space. **g** Anatomic drawings of the injection in the pelvic rectum space. **h** The Thiersch procedure was performed. **i** The results on clinical follow-up three months after surgery

Surgery was performed under spinal anesthesia, and cephalosporin was administered 0.5 h before the operation. The patient was placed in lithotomy and Trendelenburg positions to prevent the small intestine from slipping into the Douglas pouch. The prolapsed rectum was pulled out of the anus completely using Babcock forceps, and the anus was inspected using a rotating speculum anoscope. First, the rectal mucosa was transfixed by a 2/0 Vicryl thread at the 3, 6, 9, and 12 o’clock positions, 5 cm above the surface of the prolapsed mucosa. The threads were not cut and pulled in four directions (Fig. 1a). Second, while grasping the prolapsed mucosa using hemostatic forceps, the grips on the prolapsed rectal mucosa were ligated using a 3/0 silk thread to create a tag; this procedure was repeated in multiple transverse and longitudinal lines that were arranged at 0.5 cm intervals without excising the tag (Fig. 1b). Third, the sclerosant (1 mL, 50% XiaoZhiLing) was injected into the rectal submucosa proximal to the mucosal plication (Fig. 1c). Fourthly, the prolapsed rectal mucosa 1 cm above the dentate line was sutured with a previously uncut Vicryl thread 2/0 (Fig. 1d). Fifth, the prolapsed rectum was completely restored by tightening the Vicryl thread (Fig. 1e). Sixth, the index finger was placed into the rectum, and the needle was inserted 8 cm from the 1.5 cm anal verge in the 3 and 9 o’clock positions. Under the guidance of the index finger and ultrasound, the sclerosant (20 mL 100% XiaoZhiLing) was injected into the pelvic rectum space (Fig. 1f, g). Finally, four 3-mm-long vertical incisions were made in the midline of the anal verge anteriorly and posteriorly at the 3, 6, 9 and 12 o’clock positions. All the incisions were sutured using a 0 Vicryl thread to encircle the entire anal verge, and the suture was tied over an index finger (Fig. 1h).

### Statistical analyses

SPSS 22.0 statistical software was used for data analysis. The measurement data were analyzed by Student’s *t*-test and expressed as the mean + standard deviation. A value of P < 0.05 was considered statistically significant.

## Results

Between January 2016 and December 2018, a total of 34 female patients with FTRP underwent nmGMTSI. The clinical characteristics of the patients are listed in Table 1. The median age was 76.2 ± 9.14 years (range 65–87 years), while the BMI was 17.3 ± 4.5 kg/m^2^ (range 12–23) with most patients underweight. All patients complained of a mass protruding through the anus. In 85.3% of patients, the mass protruded occasionally; in 14.7% of patients, the mass protruded at all times. The initial condition in most patients was diarrhea or fecal incontinence (76.4%), while constipation was the initial presentation in 23.6% of patients. Some patients presented with abdominal or anal pain, blood discharge, and mucus discharge (Table 1). To explore the etiology of FTRP, the risk factors for FTRP are provided in Table 2. The potential risk factors analyzed were multiparity, chronic obstructive pulmonary disease, malnutrition, redundant rectosigmoid colon, previous pelvic surgery, colitis, and irritable bowel syndrome. This result was slightly different from that in Western societies.

**Table 1:** Clinical characteristics of patients undergoing nmGMTSI for full-thickness rectal prolapse

	Total population (n = 34)
Age mean ± SD (years)	76.2 ± 9.14
BMI (kg/m^2^) mean ± SD	19.3 ± 4.5
*Symptoms*	
Feeling of a bulge in the rectum during defecation	29 (85.3%)
Feelings of prolapse without defecating	1 (14.7%)
Diarrhea/Fecal incontinence	26 (76.4%)
Constipation/obstructed defecation	8 (23.5%)
Abdominal or anal pain	8 (24%)
Blood discharge	13 (38.2%)
Mucus discharge	10 (29.4%)

*SD* standard deviation, *BMI* body mass index

**Table 2:** Risk factors for full-thickness rectal prolapse

Factors	Total population (n = 34)
Vaginal delivery ≥ 3	29 (85.3%)
< 3	5 (14.7%)
Chronic obstructive pulmonary disease	23 (67.6%)
Malnutrition/long-term vegetarians	21 (61.7%)
Redundant rectosigmoid colon	18 (52.9%)
Previous pelvic surgery	13 (38.2%)
IBD or colitis	12 (35.3%)
IBS	8 (23.5%)
Solitary rectal ulcer	5 (14.7%)
Family history of rectal prolapse	4 (11.8%)

*IBD* inflammatory bowel disease, *IBS* inflammatory bowel syndrome

**Fig. 2:**
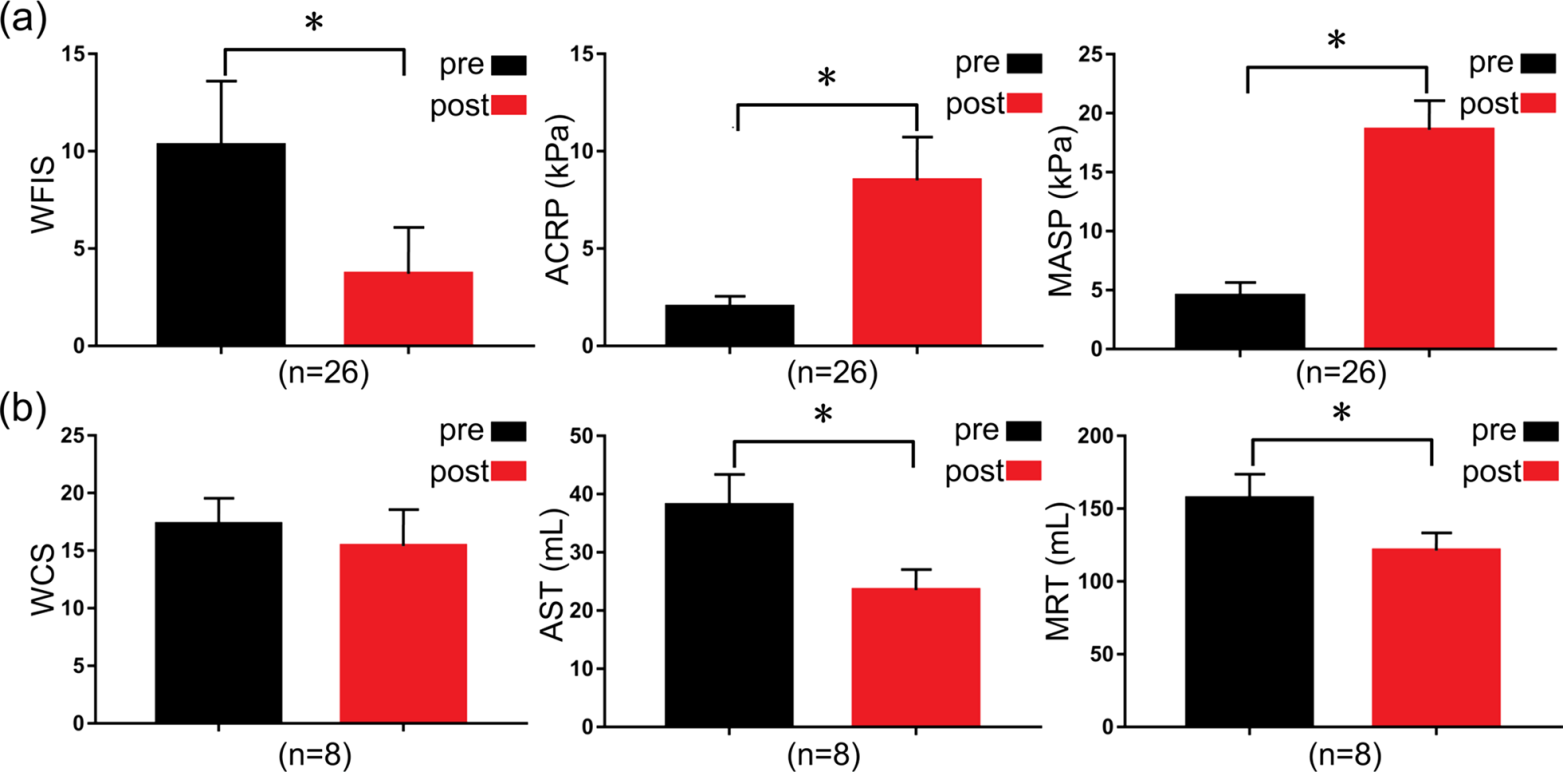
Surgical results of nmGMTSI. **a** Improvement in incontinence after nmGMTSI. WFIS, ACRP, and MASP were evaluated for patients preoperatively and postoperatively. Quantitative data (mean ± SD) were shown; *P < 0.05, Student’s *t*-test. pre: preoperative, post: postoperative. **b** Partial improvement in constipation incontinence after nmGMTSI. WCS, AST, and MRT were evaluated for patients preoperatively and postoperatively. Wexner fecal incontinence score: WFIS; anal canal rest pressure: ACRP; maximum anal systolic pressure: MASP; Wexner constipation scores: WCS; anorectal sensation thresholds: AST; maximum rectal tolerance: MRT

To analyze the causes of recurrence and complications, hospital stay, operative time and intraoperative and postoperative complications were examined (Table 3). The average hospitalization time was 7.3 ± 1.6 days (range 5–9), and most of the patients were discharged before one week. The median operative time was 44 ± 12 min (range 30–60 min). The symptoms of FTRP disappeared completely after surgery in all patients. All patients were cured according to clinical cure standard (Fig. 1i) [6]. Twenty-six patients suffered from preoperative diarrhea or fecal incontinence, which improved in 25 patients after surgery. The perioperative Wexner fecal incontinence score (WFIS) was 10.3 ± 3.31, which became 3.7 ± 2.43 (P < 0.0001) postoperatively. The perioperative and postoperative ACRP were 2.0 ± 0.56 kPa and 8.5 ± 2.25 kPa, respectively (P < 0.0001). The MASP was 4.5 ± 1.16 kPa, which became 18.6 ± 2.50 kPa postoperatively (P < 0.0001) (Fig. 2a). Eight patients experienced preoperative constipation, which improved in four patients after surgery. However, no significant difference was observed between preoperative and postoperative Wexner constipation scores (WCS) (17.3 ± 2.25 vs. 15.4 ± 2.89, P = 0.1047). The perioperative and postoperative AST were 38.1 ± 5.34 mL and 23.5 ± 3.61 mL, respectively (P = 0.0002). The perioperative and postoperative MRT were 157.1 ± 16.73 mL and 121.2 ± 12.45 mL, respectively (P = 0.0009). (Fig. 2b). Three patients (8.8%) developed rectal bleeding, which was controlled using an anal pack and conservative measures. Two patients (5.9%) showed urinary retention after surgery, which was controlled by indwelling catheters for 1 week. Two patients (5.9%) showed ischemic colitis, which was controlled by conservative measures. One patient (2.9%) developed rectal stenosis 6 months after the operation, and this was controlled by dilatation of the anus with a metal anoscope. Lastly, one patient (2.9%) showed a perianal abscess at 12 months after the operation, which was due to the infection of the thread used for the Thiersch procedure. Anti-infective therapy was ineffective, and rectal prolapse recurred one year after the infected thread had been removed.

**Table 3:** Surgical management, results, and complications

	Numbers
Hospital stays (days)	7.3 ± 1.6
Operative time (min)	44 ± 12
FTRP curative ratio	34/34 (100%)
Curative ratio of diarrhea or fecal incontinence	25/26 (96.2%)
Curative ratio of constipation	4/8 (50.0%)
Recurrence of rectal prolapse	1/34 (2.9%)
Rectal bleeding	3
Ischemic colitis	2
Urinary retention	2
Stenosis of the rectum	1
Perianal abscess	1
Infection of the thread used for the Thiersch procedure	1

*FTRP* full thickness rectal prolapse

## Discussion

There are two types of rectal prolapse as follows: Type I, which is also called mucosal prolapse, involves the protrusion of the mucosa and is usually less than 3 cm long; Type II, also called FTRP, involves full-thickness extrusion of the rectal wall characterized by concentric folds in the prolapsed mucosa [6]. FTRP commonly affects older women, and this study focused on the treatment of FTRP in older women, in whom the length of rectal prolapse is > 4 cm.

The various approaches of FTRP can be classified into two as follows: transabdominal and transperineal [3]. The choice of the initial treatment was based on the assessment, age, comorbidities, and grading of prolapse. Transperineal surgery consists of Delorme’s, Altemeier’s, STARR, and GMT operations. Delorme’s procedure involves mucosal and submucosal dissection, plication of the remaining muscle layer, and mucosal anastomosis. Delorme’s procedure has been reported to cause significant bleeding and perforation after surgery [7]. Altemeier’s procedure involves excision of the redundant rectum or sigmoid colon, and it is more appropriate for obstructive defecation syndrome [8]. It has a risk of fatal complications, such as anastomotic breakdown, rectal bleeding, and perianal abscess. This approach has been reported to have a postoperative mortality rate of 1.6% after surgery [9, 10]. The STARR operation involves excision of the redundant rectal mucosa with a colorectal anastomosis that only applies to a mucosal protrusion of less than 5 cm long [11, 12]. Since FTRP is a benign disease, the operative method should be simple with wide adaptation and low postoperative mortality rate and cost.

One surgical technique for FTRP is the GMT (mucosal plication with anal encircling), which can be used irrespective of the length of the rectum and colon prolapse [4]. Iida et al. reported no postoperative complications in 166 patients who underwent GMT. GMT is not popular in the western world, while it plays a major role in the treatment of FTRP in China and Japan [4, 5]. Possible reasons include anatomical and dietary factors. In Western societies, an elongated sigmoid colon is commonly observed in elderly institutionalized patients with chronic constipation. A redundant, elongated sigmoid colon is prone to rectal prolapse [13]. In China, the pathophysiology of FTRP is usually related to several anatomic concerns, such as obstetric trauma, causing weakness of pelvic floor muscles associated with connective tissue MMP-1(Matrix Metalloproteinases), a proteolytic enzyme involved in chronic obstructive pulmonary disease. This leads to loosely attached rectal mucosa (to the underlying muscularis) [14] (Table 2) and osteoporosis-induced malnutrition and aging, resulting in loss of the normal sacral curvature and the curvature of the rectum [15, 16]. Constipation seldom occurs partly due to the long-term vegetarian lifestyle in China [17]. Diarrhea or fecal incontinence was thought to be the common accompanying symptoms of FTRP (Table1). Clinical results of GMT showed improved defecation with minimal complications [18]. Therefore, in selecting surgical approaches, the exact causative factors and anatomical variations should be considered and tailored according to the patient’s disease characteristics. However, the recurrence rate after GMT was 23.8% within a maximum follow-up period of 14 years [4]. We modified the GMT and combined it with square transfixion with submucosal and ischiorectal space injection sclerotherapy to address this problem.

Compared to GMT, we made the following three improvements in nmGMTSI: First, the grip of the full thickness of the rectal wall was transfixed by a 2/0 Vicryl thread at the 3, 6, 9, and 12 o’clock positions, 5 cm above the surface of the prolapsed mucosa. Similar to GMT, multiple tags were created. After the rectal mucosa, the tissue 1 cm above the dentate line was sutured with the previously uncut Vicryl thread 2/0, and the prolapsed rectum was completely restored by tightening the Vicryl thread. This improvement can significantly shorten the operation time and prevent recurrence in the short term after surgery, in which inflammatory adhesions have not yet formed. Second, longitudinal injection of the sclerosant in each of the four quadrants of the rectal submucosal area promotes inflammatory response and scarring, which prevents long-term mucosal prolapse recurrence. Finally, the sclerosant was injected into the pelvic rectal space. This improvement was mainly due to the weakness of the pelvic floor muscles associated with connective tissue. The sclerosant initiates an inflammatory reaction resulting in fibrosis outside the rectal wall, and the perirectal tissue that leads to the wall of the rectum adheres to the perirectal tissue, preventing recurrence of prolapse of the rectal wall. Of course, sclerosant should be avoided in the anal sphincter.

Evaluation of the nmGMTSI depends on the results and complications of the surgery. As shown in Table 3, some of our patients had comorbidity; however, they all recovered satisfactorily from surgery. No perforation of the rectum or colon was observed. This may reflect the fact that nmGMTSI is a safe and extremely effective operating method. In this study, the WFIS was significantly lower after surgery (Fig. 2a). Our study findings are consistent with those of the study of Yamana et al. [18], as we observed improvement in incontinence in more than 96.2% of patients (Table 3). One possible explanation is that the ACRP and MASP were significantly increased after anal encircling, and the wall of the rectum adhered to the perirectal tissue (Fig. 2a). Constipation partially improved in this study (Table 3). The AST and MRT were downregulated postoperatively compared to the preoperative values. However, no significant difference was between the preoperative and postoperative WCS (Fig. 2b). One possible reason is that scar formation in the rectal submucosal area would result in outlet obstruction. As shown in Table 3, some patients developed complications, such as bleeding, colitis, and stenosis; however, they all recovered satisfactorily with conservative therapies. Notably, the thread used for anal encircling can be infected, and its removal was usually necessary. In such cases, recurrence tends to occur after removal.

In our study, the overall recurrence rate after nmGMTSI was 2.9% during a period of two years with no operative death. The nmGMTSI is effective for diarrhea or fecal incontinence. There are, of course, some limitations to this research. The postoperative follow-up time was not long enough, and the patients’ satisfaction scores were not validated. The nmGMTSI is less effective for cases of constipation or obstructed defecation.

## Conclusion

The nmGMTSI has various benefits, including wide adaptation, minimal invasiveness, technical simplicity, cost-effectiveness, and low rates of complications, mortality, and recurrence. Hence, we recommend this procedure be used as the first choice for FTRP in frail elderly women with pelvic floor muscle weakness.

## Data Availability

All data generated or analyzed during this study are available from the corresponding author on reasonable request.

## Abbreviations

FTRP: Full-thickness rectal prolapse
STARR: Stapled transanal rectal resection
GMT: Gant-Miwa-Thiersch
nmGMTSI: New modified GMT combined with internal and external rectal sclerosant injection
WFIS: Wexner fecal incontinence score
ACRP: Anal canal rest pressure
MASP: Maximum anal systolic pressure
WCS: Wexner constipation scores
AST: Anorectal sensation thresholds
MRT: Maximum rectal tolerance
IBD: Inflammatory bowel disease
IBS: Irritable bowel syndrome
